# MCT8 Deficiency in Two Brothers With a Novel Deletion Mutation in *SLC16A2*

**DOI:** 10.1155/crig/5589985

**Published:** 2025-12-15

**Authors:** Andrea A. Arcari, María Eugenia Rodríguez, Romina Armando, María Sol Ayuso, Matias T. De Iuliis La Torre, Marina Szlago

**Affiliations:** ^1^ Division of Endocrinology, Center for Endocrinological Research ‘Dr. César Bergadá’ (CEDIE), Ricardo Gutiérrez Children’s Hospital, Buenos Aires, Argentina; ^2^ Medical Genetics Section, Ricardo Gutiérrez Children’s Hospital, Buenos Aires, Argentina; ^3^ Medium and High Complexity Outpatient Clinic (CMAC), Ricardo Gutiérrez Children’s Hospital, Buenos Aires, Argentina; ^4^ Division of Pediatric Neurology, Ricardo Gutiérrez Children’s Hospital, Buenos Aires, Argentina; ^5^ Section of Rare Diseases and Inborn Errors of Metabolism, Ricardo Gutiérrez Children’s Hospital, Buenos Aires, Argentina

**Keywords:** Allan–Herndon–Dudley syndrome, delayed myelination, developmental delay, hypomyelination, MCT8 deficiency, *SLC16A2*

## Abstract

**Background:**

Monocarboxylate transporter 8 (MCT8) deficiency, also known as Allan–Herndon–Dudley syndrome, is a rare X‐linked genetic disorder resulting from pathogenic mutations in the *SLC16A2* gene. MCT8 deficiency impacts the transport of thyroid hormone (TH) throughout the body. Symptoms are highly heterogeneous and often include severe neurodevelopmental delay, hypotonia in the limbs and trunk, and low body weight.

**Case Presentation:**

This case report describes the diagnostic journey of two brothers born 1 year and 8 months apart to nonconsanguineous parents. Both exhibited severely limited motor development, quadriparesis, and an inability to sit independently or hold their head up. Despite their significant clinical presentations, the diagnoses were delayed: 12 years and 8 months for Case 1 and 8 years and 3 months for Case 2. Genetic testing revealed that both patients carry the same novel *SLC16A2* mutation, 960_995del (p.Tyr321_Ala332del). Although they displayed key clinical features of MCT8 deficiency, the TH profile of Case 1 was inconclusive, lacking the characteristic sustained elevation of triiodothyronine (T3) typically associated with MCT8 deficiency.

**Conclusions:**

Findings from this case report highlight the need for greater awareness of this disorder and underscore the clinical heterogeneity of MCT8 deficiency.

## 1. Introduction

Monocarboxylate transporter 8 (MCT8) is a critical and highly specific thyroid hormone (TH) transporter encoded by the solute carrier family 16, member 2 (*SLC16A2,* OMIM #300095) gene [1, 2]. Mutations in *SLC16A2* cause the rare, X‐linked genetic disorder MCT8 deficiency, also known as Allan–Herndon–Dudley syndrome (AHDS) [3]. MCT8 deficiency primarily affects males, although a few confirmed cases have been reported in females [4, 5]. The disorder has a prevalence of fewer than one in one million based on diagnosed cases to date [6]. A broad spectrum of inherited and *de novo* pathogenic mutations has been identified in *SLC16A2,* including large deletions, frameshift deletions, single amino acid deletions, insertions, and substitutions [3, 7–10].

TH is essential for the development and function of tissues throughout the human body [11, 12]. Individuals with MCT8 deficiency typically exhibit a characteristic TH profile: serum thyroid–stimulating hormone (TSH) within the normal age‐specific range, normal to low serum thyroxine (T4), and elevated serum triiodothyronine (T3) [3, 7, 13]. The precise mechanism underlying this unique TH profile in MCT8 deficiency is yet to be fully elucidated. It is hypothesized that the loss of MCT8 function alters the sensitivity of the pituitary gland and hypothalamus to TH, reduces T4 secretion from the thyroid, and increases T4 “trapping” in the kidneys, ultimately leading to elevated serum T3 levels [7].

In the brain, TH transport into neurons is highly MCT8 dependent. However, in tissues outside the brain, TH uptake can occur through alternative transporters, albeit with lower specificity [8]. This results in two distinct but concurrent sets of symptoms: low TH levels in the brain and peripheral thyrotoxicosis due to elevated T3 levels in tissues outside the brain. Neurological symptoms may include hypotonia in the head and trunk, dystonia in the arms and legs, seizures, lack of speech development, and unclear cognitive development. Symptoms of peripheral thyrotoxicosis can include low weight, muscular atrophy, and cardiovascular abnormalities. However, the clinical presentation of MCT8 deficiency is highly heterogeneous [6, 9, 14].

In most cases, pregnancy and the first few months of life are uneventful in patients with MCT8 deficiency. The first presentation of an individual with MCT8 deficiency to a healthcare professional is typically related to global developmental delay or failure to thrive [9]. Over time, symptoms become increasingly evident, prompting further diagnostic evaluation. Despite the severity of symptoms, the broad range of potential differential diagnoses and limited awareness of MCT8 deficiency often contribute to delayed diagnosis [15].

This case report describes the diagnostic journey and clinical presentation of two brothers with MCT8 deficiency, both carrying the same mutation in *SLC16A2*. Despite the severity of symptoms, there was a significant delay between symptom onset and diagnosis. These patients did not, and do not, consistently exhibit the typical TH profile (high T3 and low to normal T4/TSH) commonly associated with MCT8 deficiency.

Despite sharing the same deletion mutation and some clinical characteristics, specific aspects of the brothers’ phenotypes differ. This highlights the need for greater awareness of the disorder and underscores the potential heterogeneity in clinical presentation among individuals with MCT8 deficiency, even those with the same pathogenic variant.

## 2. Case Description

The patients described in this report are brothers born to nonconsanguineous parents. Case 1 initially presented with irritability and food rejection at 1 month of age, followed by global developmental delay at 5 months. Case 2 was born 1 year and 8 months later, with developmental delay noted at 5 months of age. In 2015, both patients were referred to the Section of Rare Diseases and Inborn Errors of Metabolism and the Center for Endocrinological Research “Dr. César Bergadá” (CEDIE) at the Ricardo Gutiérrez Children’s Hospital by their pediatrician due to global developmental and altered thyroid and adrenal function, respectively.

### 2.1. Case 1

Case 1 is a 13‐year‐old male patient. He was born prematurely at 35 weeks, with a birth weight of 2040 g (3rd–10th percentile). He was admitted to inpatient care for 15 days for nutritional recovery and later received a normal neonatal screening result. At 1 month of age, irritability and food rejection were reported. Between five and seven months of age, he was hospitalized in the general pediatric department of the hospital for further testing due to neurodevelopmental delay.

In 2015, at 3 years and 10 months of age, upon referral to the endocrinology department, his weight was 10.5 kg (< 3^rd^ percentile), height was 95 cm (3rd–10th percentile), and cranial circumference was 44 cm (< 3^rd^ percentile). He exhibited generalized muscle stiffness and thumb inclusion. His heart rate was within the normal range (87 beats per minute [bpm]). The thyroid gland was not palpable, and laboratory testing was requested to confirm cortisol levels and thyroid profile; however, follow‐up was not completed.

He returned for consultation in 2018, at 6 years and 6 months of age. Laboratory testing revealed TSH within normal range (4.52 µUI/mL), low free T4 (0.57 ng/dL), and low cortisol (4.2 μg/dL). He was diagnosed with hypothyroidism and pituitary insufficiency, and treatment with levothyroxine (LT4) at 25 mcg/day and hydrocortisone at 12 mg/m^2^/day was initiated. Stress management guidelines were also provided to the caregivers. Follow‐up during this period was irregular; however, over 19 months, LT4 dosing was gradually increased to 112 mcg/day to achieve free T4 within the reference range. At 7 years of age, his weight was 13.9 kg (< 3^rd^ percentile) and his height was 112 cm (3^rd^ percentile).

Following the COVID‐19 pandemic, visits to the endocrine specialists were resumed at age 10 years and 4 months in April 2022. He had remained on LT4 at a dose of 112 mcg/day, and hydrocortisone had been reduced to 7.3 mg/m^2^/day. The hydrocortisone dose was adjusted to 8.8 mg/m^2^/day, and treatment with melatonin was initiated due to reported issues with sleep.

A diagnosis of MCT8 deficiency was confirmed in May 2022 at 12 years and 8 months of age. TH measurements recorded after the diagnosis of Case 1 are shown in Table 1.

**Table 1:** TH profile of Case 1.

	April 2023	September 2023	November 2023	November 2023	December 2023	April 2024	December 2024	February 2025
TSH (μUI/mL)	< 0.01	< 0.01	< 0.01	26.4	1.1	1.37	0.02	4.06
Free T4 (ng/dL)	1.23	1.13	0.91	0.22	0.82	0.72	0.88	0.83
Total T3 (ng/dL)	—	371	300	—	244	—	353	159.5
LT4 dose (mcg/day)	112	Suspended	Suspended	75	75	88	50	50

*Note:* Measurements recorded after diagnosis of MCT8 deficiency and regular inclusion of total T3 level. Dose adjustments of LT4 at each assessment are included.

Following laboratory test results in September 2023 showing low TSH (< 0.01 µUI/mL), free T4 within the reference range (1.13 ng/dL), and high total T3 (371 ng/dL), LT4 treatment was suspended. Repetition of testing over the following two months showed a progressive decline in free T4, and LT4 treatment was resumed in November 2023 at a dose of 75 mcg/day. Laboratory results 1 month later showed TSH, free T4, and total T3 within the normal range. The LT4 dose was once again increased to 88 mcg/day in April 2024 due to low free T4 levels.

In December 2024 (age 13), his TH profile again showed suppressed TSH, T4 within the reference range, and high T3. At this assessment, the LT4 dose was reduced to 50 mcg/day, with treatment withdrawal planned, and alternative treatment with tiratricol (Emcitate) was considered but not initiated. In February 2025, TSH, free T4, and total T3 were within the reference ranges.

Case 1 has no motor development, quadriparesis, and is unable to sit independently. He has not developed speech but demonstrates the ability to make a visual connection with a social smile. Irritability was noted as early as 1 month of age, and agitation has been reported since then; however, this appears to have improved with age.

Surgical interventions have included the correction of bilateral hip dislocation and bilateral cryptorchidism (both at 12 years of age), with no reported complications in these procedures.

### 2.2. Case 2

This case is the younger brother of Case 1. He was born at 39 weeks via vaginal delivery following a controlled pregnancy, and neonatal screening results were normal. His birth weight was 3700 g but progressively declined with age. Developmental delay was noted at 5 months of age.

He began developing infantile spasms at 9 months of age, and an electroencephalogram (EEG) showed hypsarrhythmia. Symptoms were initially controlled with valproic acid, vigabatrin, and corticosteroids. At 3 years of age, another EEG showed poor sleep organization. Seizures persisted, and he was subsequently diagnosed with refractory epilepsy, showing no response to anticonvulsant drugs (rufinamide, levetiracetam, lamotrigine, and clonazepam) or a ketogenic diet.

In 2018, at 5 years and 4 months of age, TH assessment revealed a low free T4 level of 0.58 ng/dL and TSH within the reference range (2.48 µUI/mL). Antibodies against antithyroid peroxidase and antithyroglobulin were negative. Additionally, a marked elevation in sex hormone–binding globulin (SHBG) was recorded (> 200.0 nmol/L; reference range: 40–120 nmol/L). His weight was 15.8 kg (3^rd^ percentile), and his height was 115.5 cm (25^th^ percentile).

Upon referral to the endocrinology specialist in 2018, repeat laboratory testing confirmed previous results, with TSH measured at 2.29 µUI/mL and free T4 at 0.56 ng/dL. It was noted that the thyroid gland was not palpable, and his heart rate was 80 bpm. Generalized muscle stiffness and spasms, with thumb inclusion, were also recorded. He was diagnosed with central hypothyroidism, and treatment with LT4 50 mcg/day was initiated. He required a progressive increase in the LT4 dose to 100 mcg/day to normalize free T4 levels.

At 8 years of age, he exhibited 3 months of recurrent vomiting. Decreased gastric capacity was identified and attributed to scoliosis. Symptoms were managed with omeprazole and diet fractionation. A video swallowing test revealed worsening oronasal reflux compared with previous assessments, and the placement of a gastric tube to aid feeding was recommended but not placed.

In 2022, after the COVID‐19 pandemic, at 8 years and 4 months of age, he returned for an endocrinology follow‐up, having remained on LT4 treatment at 100 mcg/day. Laboratory results showed suppressed TSH (≤ 0.01 µUI/mL) with free T4 and total T4 within the reference range. Cortisol was low at 5.4 μg/dL, and treatment with hydrocortisone at a physiological dose was indicated.

Case 2 passed away during hospitalization for a respiratory infection at 9 years of age.

Case 2 showed quadriparesis and no motor development. He was unable to sit or control his head independently and required full support. He exhibited hypotonia in the trunk and limbs, along with dystonic movements. He had poor, intermittent visual connection and no signs of agitation.

### 2.3. Diagnosis

**Figure 1:**
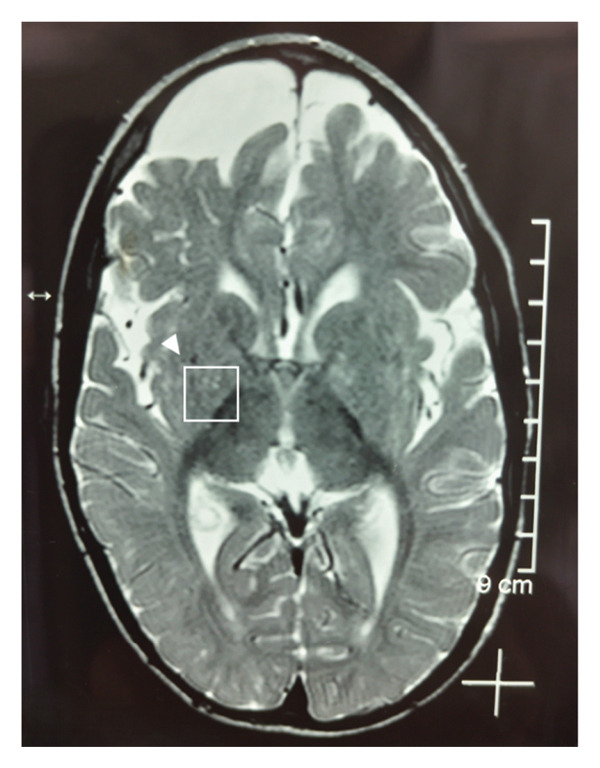
Case 1: Axial T2‐weighted MRI image at 5 years, 11 months of age. White box and arrowhead indicate region of interest in the lentiform nucleus. Confluent and diffuse hypomyelination was confirmed.

In 2017, when Case 1 was 5 years and 11 months of age and Case 2 was 4 years and 3 months of age, magnetic resonance imaging (MRI) evaluation revealed confluent and diffuse cerebral hypomyelination in both cases. An axial T2‐weighted MRI image for Case 1 showing a region of interest in the lentiform nucleus is presented in Figure 1. Based on MRI findings, diagnoses of Pelizaeus–Merzbacher–like disease or mitochondrial encephalopathy were initially strongly suspected. Other inherited autosomal recessive white matter diseases were also considered. They were subsequently referred by pediatric neurology to the Section of Rare Diseases and Inborn Errors of Metabolism. After evaluation, comprehensive genetic testing was initiated using the Invitae Leukodystrophy and Genetic Leukoencephalopathy Panel (Invitae #55002). This test included sequence analysis and deletion/duplication testing of 697 genes, including *SLC16A2*.

Both cases were found to carry a c.960_995del (p.Tyr321_Ala332del) mutation in *SLC16A2,* and a diagnosis of MCT8 deficiency was confirmed. This mutation in exon 3 results in the deletion of 12 amino acids but preserves the integrity of the reading frame. Further investigation using the Variant Effect Predictor program suggests this in‐frame deletion has a “moderate” impact on function [16]. The deleted region (Tyr321–Ala332) is also highly conserved across vertebrates, including mammals, birds, and reptiles in the UCSC Genome Browser [17]. This is the first report of this specific mutation in MCT8 deficiency patients, although mutations in this region of the gene have previously been shown to be pathogenic for MCT8 deficiency [10, 18].

The two patients were diagnosed simultaneously in May 2022, resulting in a diagnostic delay of 10 years and 5 months for Case 1 and 8 years and 3 months for Case 2, respectively. It was noted that the mother has two deceased brothers who displayed very similar clinical manifestations to the cases described in this report. One maternal uncle passed away within the first year of life, and the other at 11 years of age. Both exhibited movement disorders and profound developmental delay, resembling the two cases described here. While no DNA was available for the testing of the maternal uncles, and genetic testing of the mother is not possible due to financial constraints, there is now a high suspicion that these cases were also the result of MCT8 deficiency.

## 3. Discussion

This report presents the case history of two brothers who received a delayed diagnosis of MCT8 deficiency, despite Case 1 displaying symptoms that were retrospectively indicative of the disorder as early as 1 month of age. Although more recent data indicate that the diagnostic interval for patients with MCT8 deficiency is shortening, some patients, like the brothers described in this report, are still left waiting for over 10 years for a diagnosis. Earlier diagnosis can provide the opportunity for early intervention and specialized supportive care, such as the movement and sitting aids, nutritional support, and surgical resolution for scoliosis received by both brothers. Supportive interventions such as these can enhance the quality of life for both patients and caregivers [9, 19].

A diagnosis of MCT8 deficiency in these patients was not considered prior to receiving the genetic screening report. However, they exhibited some of the key symptoms of the disorder, including typical motor, developmental, and MRI findings attributable to MCT8 deficiency [9, 20]. Patients with MCT8 deficiency are typically born at full term after an uneventful pregnancy, with normal weight. While Case 2 was born at full term and within the normal weight range, Case 1 was born at 35 weeks with a birth weight smaller than expected for his gestational age. After a brief period of inpatient nutritional recovery, Case 1 received normal neonatal screening results. Both cases then exhibited the typical decline in expected body weight for age, commonly seen in patients with MCT8 deficiency. They initially presented to their pediatrician with developmental delay, which is the most common reason for the first presentation of individuals with MCT8 deficiency to their healthcare professional. Both brothers displayed markedly limited motor development, with quadriparesis, axial hypotonia, and an inability to hold their heads or sit independently. Furthermore, upon MRI imaging, both brothers showed delayed cerebral myelination, the most common MRI finding in a large natural history study of individuals with confirmed MCT8 deficiency [9, 20]. Although these symptoms collectively point to MCT8 deficiency, the potential for differential diagnosis remains broad. X‐linked and autosomal recessive genetic disorders, such as Pelizaeus–Merzbacher–like disease or mitochondrial encephalopathy, present similarly and are more common than MCT8 deficiency. A greater awareness of MCT8 deficiency amongst clinicians will lead to the consideration of this disorder earlier in the diagnostic journey, alongside more common disorders. With the increasing accessibility of broad genetic sequencing assays, the inclusion of *SLC16A2* in a diagnostic panel for patients with these nonspecific symptoms should be considered, despite the low prevalence of the disorder [15].

A lack of awareness of MCT8 deficiency within the healthcare community likely contributed to the extended time between symptom onset and diagnosis in these patients [15, 19]. A high degree of heterogeneity in the symptomatic presentation of MCT8 deficiency is also a key factor contributing to under‐recognition of the disorder among healthcare professionals [9, 20]. This heterogeneity is evident in the two patients described in this report, who carry the same genetic mutation in *SLC16A2* but exhibit differences in their symptomatic presentation. Seizures, which can occur in patients with MCT8 deficiency, were present in Case 2 but not in Case 1 [9]. Case 2 also showed recurrent vomiting and oronasal reflux, which may have necessitated the placement of a gastric tube. In contrast, Case 1 has not presented with either of these symptoms; however, unlike Case 2, he exhibited agitation and irritability from as early as the newborn period. Case 1 is able to make visual connections and has a social smile. In Case 2, visual connection was limited and intermittent. These differences, even between individuals with the same genetic mutation, highlight the challenges in defining a specific presentation of MCT8 deficiency. This also supports the need for individualized, dynamic care plans for patients with MCT8 deficiency, provided by an integrated multidisciplinary care team tailored to each patient’s unique symptomatic presentation [19].

Interestingly, Case 1 shows an inconclusive TH profile atypical for an individual with MCT8 deficiency. There appear to be only transient elevations in total T3 levels and consistently low T4 levels in the absence of LT4 treatment. While patients with MCT8 deficiency may show slight elevations in TSH and normal to low T4, a large retrospective cohort study found that 95% of the patients (*n* = 96/101) had a median serum T3 concentration exceeding the age‐specific upper limit [9]. Although this distinctive elevation in T3 is a common clinical finding in MCT8 deficiency, patients with T3 levels within the reference range have been reported in the literature. For example, one case report presented a patient with developmental delay, hypotonia, and delayed myelination, whose serum T3 concentration was within the reference range. A diagnosis of MCT8 deficiency was suspected based on the MRI findings of delayed hypomyelination, and the patient was found to carry a novel mutation in exon 3 (p.L291R) of *SLC16A2* [21]. It has been shown that pathogenic mechanisms in MCT8 deficiency may vary depending on the specific mutation in *SLC16A2*. Different mutations may, therefore, impact the TH profile of the individual, influencing the level of residual MCT8 transporter activity or the degree of type 1 deiodinase activity [8, 22]. Other explanations for an atypical or inconclusive TH profile include a failure to consider age‐specific ranges, comedication with hydrocortisone, and assay variability and molecular cross‐reactivity [23–27]. This case report further underscores the importance of including *SLC16A2* in genetic screening panels for patients with nonspecific developmental delay and myelination abnormalities, even in the absence of elevated T3.

Due to the extended diagnostic period for these patients, many of the investigations and interventions described in this case report were carried out before the diagnoses of MCT8 deficiency were confirmed. Managing T4 levels in both cases proved challenging, with multiple necessary adjustments and interruptions in LT4 dosing. Treatment with LT4 is associated with suppression of TSH and elevation of T3, which may exacerbate symptoms of peripheral thyrotoxicosis in patients with MCT8 deficiency [28]. This is observed in Case 1, who shows elevations in T3 only when the LT4 dose is high. Block‐replacement therapy has been considered as a treatment option for MCT8 deficiency, combining LT4 with propylthiouracil (PTU; type 1 deiodinase inhibitor) in an attempt to decrease endogenous TH production and prevent the conversion of LT4 to T3. However, PTU has been associated with neutropenia and hepatotoxicity, carrying an FDA black box warning and a contraindication for use in pediatric patients [6, 15]. Thyromimetic drugs under investigation include 3,5‐diiodothyropropionic acid (DITPA) and 3,3′,5‐triiodothyroacetic acid (tiratricol). These compounds have shown promise in reducing the endocrinological effects of thyrotoxicosis by normalizing T3 levels [14, 20, 29]. In February 2025, the European Commission granted authorization for Emcitate (tiratricol) as the first and only treatment for peripheral thyrotoxicosis in patients with MCT8 deficiency [30]. Initiating tiratricol treatment was considered for Case 1; however, at his most recent assessment, all TH markers, including T3, were within the normal range. This further illustrates the complexity of endocrinological management in patients with MCT8 deficiency, requiring constant monitoring and adding to the high burden of care for these patients.

## 4. Conclusion

MCT8 deficiency is a rare, severely debilitating, and life‐limiting disorder, with approximately 30% of the affected individuals dying during childhood. Despite ongoing improvements in recognition of the disorder, knowledge gaps within healthcare systems still lead to delayed diagnosis for many patients [15, 20].

In this case report, we describe the clinical presentation of two brothers with MCT8 deficiency, both carrying a novel deletion mutation in *SLC16A2* (c.960_995del; p.Tyr321_Ala332del). Both cases initially showed normal neonatal screening results, followed by delays in achieving developmental milestones, difficulty gaining weight, and axial hypotonia with an inability to lift their heads. These cases highlight the heterogeneity of symptoms and long‐term sequelae associated with MCT8 deficiency, with noticeable differences in phenotype observed between individuals with the same mutation and environmental influences. This report further underscores the importance of considering MCT8 deficiency as a diagnosis in individuals with delayed myelination, developmental and motor delay, and low body weight, even when the TH profile does not conclusively indicate MCT8 deficiency.

## Consent

Written informed consent was obtained from the caregiver for the publication of this case report and the accompanying images.

## Disclosure

All authors have reviewed the final manuscript and agree to be accountable for all aspects of the work presented. The funder Egetis Therapeutics was not involved in the study design, collection, analysis, interpretation of data, the writing of this article, or the decision to submit it for publication.

## Conflicts of Interest

The authors declare no conflicts of interest.

## Author Contributions

Andrea A. Arcari: contributed to manuscript preparation, review, and editing at every draft stage and provided materials, patients, or tools for manuscript development.

María Eugenia Rodríguez: contributed to manuscript preparation, review, and editing at every draft stage and provided materials, patients, or tools for manuscript development.

Romina Armando: contributed to manuscript preparation, review, and editing at every draft stage and provided materials, patients, or tools for manuscript development.

María Sol Ayuso: contributed to manuscript preparation, review, and editing at every draft stage and provided materials, patients, or tools for manuscript development.

Matias T. De Iuliis La Torre: contributed to manuscript preparation, review, and editing at every draft stage and provided materials, patients, or tools for manuscript development.

Marina Szlago: responsible for study conceptualization, methodology design, data collection, and curation; provided materials, patients, or tools for manuscript development; contributed to manuscript preparation, review, and editing at every draft stage and table and image visualization; and provided project oversight, management, and leadership.

## Funding

The authors declare that financial support was received for the research, authorship, and/or publication of this article. Editorial support for manuscript research, preparation, collation of author feedback, and journal submission was provided by Prescript Communications Ltd and funded by Egetis Therapeutics.

## Data Availability

The data used in this publication are available from the corresponding author upon reasonable request.
